# Identification and characterisation of a novel adhesin Ifp in *Yersinia pseudotuberculosis*

**DOI:** 10.1186/1471-2180-11-85

**Published:** 2011-04-28

**Authors:** Philippa CR Strong, Stewart J Hinchliffe, Hannah Patrick, Steve Atkinson, Olivia L Champion, Brendan W Wren

**Affiliations:** 1 Pathogen Molecular Biology Unit, Department of Infectious and Tropical Diseases, London School of Hygiene and Tropical Medicine, London, WC1E 7HT, UK; 2 School of Molecular and Medical Sciences, Queens Medical Centre, University of Nottingham, Nottingham, NG7 2UH, UK; 3 School of Biosciences, University of Exeter, Geoffrey Pope Building, Stocker Road, Exeter, EX4 4QD, UK

## Abstract

**Background:**

In order to identify new virulence determinants in *Y. pseudotuberculosis *a comparison between its genome and that of *Yersinia pestis *was undertaken. This reveals dozens of pseudogenes in *Y. pestis*, which are still putatively functional in *Y. pseudotuberculosis *and may be important in the enteric lifestyle. One such gene, *YPTB1572 *in the *Y. pseudotuberculosis *IP32953 genome sequence, encodes a protein with similarity to invasin, a classic adhesion/invasion protein, and to intimin, the attaching and effacing protein from enteropathogenic (EPEC) and enterohaemorraghic (EHEC) *Escherichia coli*.

**Results:**

We termed YPTB1572 Ifp (Intimin family protein) and show that it is able to bind directly to human HEp-2 epithelial cells. Cysteine and tryptophan residues in the C-terminal region of intimin that are essential for function in EPEC and EHEC are conserved in Ifp. Protein binding occurred at distinct foci on the HEp-2 cell surface and can be disrupted by mutation of a single cysteine residue at the C-terminus of the protein. Temporal expression analysis using *lux *reporter constructs revealed that *ifp *is expressed at late log phase at 37°C in contrast to invasin, suggesting that Ifp is a late stage adhesin. An *ifp *defined mutant showed a reduction in adhesion to HEp-2 cells and was attenuated in the *Galleria mellonella *infection model.

**Conclusion:**

A new *Y. pseudotuberculosis *adhesin has been identified and characterised. This Ifp is a new member in the family of invasin/intimin outer membrane adhesins.

## Background

Within the genus *Yersinia *there are three human-pathogenic species; *Y. pestis*, the causative agent of plague, and two enteric pathogens, *Y. pseudotuberculosis *and *Y. enterocolitica*. Despite the differences in disease, *Y. pestis *and *Y. pseudotuberculosis *are very closely related at the genetic level. *Y. pestis *is believed to have evolved from *Y. pseudotuberculosis *between 1,500-20,000 years ago [1]. Thus, in a remarkably short length of evolutionary time, *Y. pestis *has evolved from an enteropathogen, to a blood-borne pathogen with an insect vector [2]. Genome sequencing of several *Y. pseudotuberculosis *and *Y. pestis *strains, revealed that *Y. pestis *has accumulated a large number of pseudogenes since its divergence. By the "use it or lose it" paradigm, this is suggestive of the decay of those genes that are no longer required for function as *Y. pestis *adapts to a new lifestyle [3,4]. Gene disruption may also result in pathoadaptive mutation, whereby loss of gene function results in an increase in virulence [5]. This has been demonstrated in several pathogenic bacteria including *Shigella *spp. and *Escherichia coli *[6,7]. Pathoadaptive mutations have previously been identified in *Y. pestis*, with the negative regulators of biofilm formation, *rcsA *and *nghA*, being disrupted, resulting in the ability of *Y. pestis *to form biofilms within the flea vector [8,9].

Pseudogenes in *Y. pestis *that are known to be essential for the enteric lifestyle of *Y. pseudotuberculosis*, include the adhesins YadA and invasin [3,10,11]. Invasin was one of the first bacterial virulence factors identified, when it was observed that the *inv *gene alone was sufficient to convert benign non-invasive laboratory *E. coli *strains, to being capable of invading tissue culture cells [12]. Invasin is a 103 kDa protein that is capable of binding to β1 integrins on the host cells, promoting internalisation of the bacterium [13]. During early infection, invasin specifically binds β1 integrins on the apical surface of M cells, which facilitates efficient translocation to the underlying Peyer's patches [14]. The invasin protein is composed of a short N-terminal transmembrane domain, four structural bacterial immunoglobulin domains (bIg domains) and a C-type lectin-like domain [15]. The last bIg domain and the C-type lectin-like domain comprise the functional β1 integrin binding region [15,16].

In the same family of bacterial adhesion proteins as invasin, is intimin, an important adhesin expressed by enteropathogenic (EPEC) and enterohaemorrhagic (EHEC) *E. coli *on the LEE pathogenicity island [17]. Intimin is a 94 kDa outer membrane protein that is also found in *Citrobacter freundii *and *Hafnia alvei *[17,18]. The functional binding domain of intimin is located in the 280 amino acid C-terminal region, and consists of two bIg domains and a C-type lectin-like domain, which are structurally similar to invasin [15,18,19]. When EPEC and EHEC colonise the small intestine mucosa, a characteristic attaching and effacing lesion is formed. First there is localised destruction (effacement) of the microvilli, which leads to intimate attachment of the bacterium to the host cell [20]. EPEC and EHEC encode a specific intimin receptor, translocated intimin receptor (Tir). This receptor is translocated directly into the host cells via a type III secretion system, where it becomes expressed on the cell surface [21,22]. Intimin binds to Tir leading to its activation, which results in actin polymerisation within the host cell and the formation of a pedestal, facilitating tighter adherence between the host cell and the bacterium [17,23]. Other eukaryotic receptors have been suggested for intimin, including nucleolin and some β1 integrins, but as yet it is unknown if these interactions have a role *in vivo *[24,25].

There is considerable sequence variation between the intimins from different *E. coli *strains and they have been categorised into different subtypes, each with a high affinity for its own cognate Tir [26]. However, despite this diversity, it has been found that within the C-terminal binding domain there are four tryptophan residues and two cysteine residues, which are conserved between all subtypes [27,28]. The two cysteines are also conserved in similar locations within the *Y. pseudotuberculosis *invasin. In both invasin and intimin a disulphide bond is formed, which is essential for the structure of the C-terminal binding domain and therefore required for full functionality [29,30]. In the instance of invasin, disruption of either cysteine results in an inability to bind to integrin, and therefore is defective for invasion [29].

Analysis of *Y. pseudotuberculosis *strain IP32953 sequence data identified a gene encoding a protein with significant amino acid similarity to invasin and intimin, which has not been previously investigated. We have termed this protein Ifp (intimin family protein) and intriguingly it has been mutated to a pseudogene in all seven *Y. pestis *genomes sequenced to date. Examination of the amino acid sequence of Ifp revealed that three of the four tryptophans and both of the cysteine residues that are important in intimin function are conserved. However, no Tir orthologue can be identified in the IP32953 genome sequence. Given the amino acid similarity of Ifp to both invasin and intimin, coupled with it being putatively non-functional in *Y. pestis*, we postulated that Ifp may be an adhesin. We demonstrate that Ifp is a functional adhesin that binds to distinct foci on host cells. Expression occurs in late log or early stationary phase at 37°C only and coincides with a decline in the expression of invasin at this temperature.

## Methods

### Strains used and culture conditions

All *Y. pseudotuberculosis *strains were cultured in Luria-Bertani (LB) broth Miller (BD Biosciences, Oxford, UK) or on LB agar (Novagen, Nottingham, UK) at 28°C unless otherwise stated. The retention of the virulence plasmid (pYV) was screened by plating *Y. pseudotuberculosis *strains on CRMOX plates [31]. Antibiotics were used at the following concentrations where appropriate, ampicillin (100 μg ml^-1^), kanamycin (50 μg ml^-1^), chloramphenicol (30 μg ml^-1^) and trimethoprim (100 μg ml^-1^). *E. coli *strains were cultured at 37°C overnight in LB broth Miller or on LB agar unless otherwise stated. See table 1 for a list of the strains and plasmids used in this study.

**Table 1:** The strains and plasmids used in this study

Strain or plasmid	Reference
*Y. pseudotuberculosis *IP32953	[3]
*Y. pseudotuberculosis *IP32953 ΔIFP	This study
*Y. pseudotuberculosis *IP32953 ΔINV	This study
*Y. pseudotuberculosis *IP32953 ΔIFPΔINV	This study
*Y. pseudotuberculosis *IP32953 ΔIFPpIFP	This study
*Y. pseudotuberculosis *IP32953 *YPTB1572*Lux	This study
*Y. pseudotuberculosis *IP32953 *YPTB1668*Lux	This study
*E. coli *TB1 MBP-Ifp	This study
*E. coli *TB1 MBP-Ifp_C337G_	This study

### Construction of *lux *reporter strains

PCR primers (Table 2) were designed to amplify 956 bp and 636 bp fragments between *YPTB1572 *and *YPTB1573 *and between *YPTB1667 *and *YPTB1668 *respectively using *Y. pseudotuberculosis *strain IP32953 genomic DNA as a template. These regions contain the putative promoter and regulatory sequences for *ifp *(*YPTB1572*) and *inv *(*YPTB1668*). These PCR products were cloned into the pGEM-T Easy vector (Promega, Southampton, UK). *Kpn*I and *Spe*I restriction sites had been incorporated into the primer sequences to enable the *lux*CDABE operon from pBlue*lux *[32] to be inserted downstream of each promoter region. The entire promoter-*lux *construct was excised from pGEM-T Easy then re-cloned into the pDM4 suicide plasmid using Transformax EC100D *pir*+ *E. coli *(Epicentre Biotechnologies, Madison, USA) for selection and screening. The resulting promoter fusions *1572*lux and *1668*lux in pDM4 were then electroporated into the IP32953 strain of *Y. pseudotuberculosis *and screened for single crossover event into the genome by chloramphenicol resistance. This crossover event resulted in a functional gene of interest, with the *lux *cassette with native promoter inserted upstream of the gene on the chromosome.

**Table 2:** Primers used in this study

Primer	Sequence
YPTB1572Lux1	TTT**CCCGGG**CACCTTGGCTGCACCGACTTC
YPTB1572Lux2	TTT**GGTACC**CGATAGAGACTCATACTTACC
YPTB1668Lux1	TTT**CCCGGG**CATTTTGGGTGAACACAGAGG
YPTB1668Lux2	TTT**GGTACC**GAGAAACTCACTGATTGGCTG
YptbIntMBP-1	TCA**GAATTC**ATTAGTGAAGTCACCCCAAC
YptbIntMBP-2	TCA**TCTAGA**TGTGCCAGAGCCCTCCTAACC
YptbIntMBP-3	TCA**TCTAGA**TTTATTTTATACCCATGTAAAGC
INTPROM3	TTT**GGTACC**TCAATTACATATCGTTAACGC
INTPROM4	TTT**GCATGC**GATCTGTCTAAAGAGCGTCG
INTA	TTTGCATGCTGGAGTATAGGTAAGTATGAG
INTB	TTTGAGCTCGTTTGCACATCGGCTAATGG
YPTB1668Chlor1	CAGGTCCAGCCTTATTCTGTCTCTTCATCTGCATTTGAAAATCTCCATCCTCACTTATTCAGGCGTAGCAC
YPTB1668Chlor4	CGTTCTCCAATGTACGTATCCCGACGCCAAGGTTAAGTGTGTTGCGGCTGCATAGTAAGCCAGTATACACTC

Restriction sites are in bold and position of mutated cysteine to glycine residue is underlined.

### Bioluminescence and optical density of Lux constructs

Bioluminescence and optical density were monitored simultaneously in 96-well microtitre plates using the Anthos Lucy1 combined photometer and luminometer controlled by the Stingray software (Dazdaq). Overnight 30°C cultures of *1572lux *and *1668lux *were diluted 1:200 in fresh LB with antibiotics and grown for 3-4 h at 30°C. The optical density of the culture was adjusted to a starting OD_600 _of 0.1 and 200 μl was added to the wells of the 96 well plate. Assays were performed at 24°C, 28°C and 37°C. Luminescence and OD at 405 nm of the cultures was automatically determined every 30 min for 18 h and presented as relative light units per unit of OD_405 _(light per unit cell). For every promoter fusion assay each sample was assayed in triplicate on the 96-well plate, and each experiment was carried out in duplicate.

### Construction of Maltose Binding Protein (MBP) fusion proteins

Primers YptbIntMBP-1 and YptbIntMBP-2 (Table 2) were used to PCR amplify the 3' end (1044 bp) of the *ifp *coding sequence. This PCR product was cloned into the pMAL-p2x vector (NEB, Hitchin, UK) using *Eco*RI and *Xba*I enzyme restriction sites which had been incorporated into the primer design. In order to generate an MBP-Ifp fusion with the terminal cysteine (Cys1070) mutated to a glycine (MBP-Ifp_C337G_), primer YptbIntMBP-3 was substituted for YptbMBP-2. Primer YptbIntMBP-3 contained an alternative sequence (underlined in table 2) to mutate the terminal cysteine to a glycine. MBP-fusion proteins were then expressed in TB1 *E. coli*.

### Purification of MBP-Ifp and MBP-Ifp_C337G_

*E. coli *transformed with the MBP-fusion plasmids were cultured for 2 h at 37°C using 5 ml of an overnight culture in 250 ml LB broth with 2 mM glucose and ampicillin until a culture growth of OD_600 _0.5 was reached. Expression of the fusion protein was induced with 0.3 mM isopropyl-β-D-thiogalactoside (IPTG) then the culture was incubated for further 2 hours at 37°C. The cultures were centrifuged and pellets stored overnight at -20°C then resuspended in column buffer (20 mM Tris-HCl, 200 mM NaCl, 1 mM EDTA in H_2_O). The cells were lysed by sonication using a Bioruptor sonicator (Diagenode; 60 second pulses with a 30 second recovery period). Insoluble proteins were removed by centrifugation and the supernatant was applied to 1 ml columns of amylose resin (NEB). After washing with 15 ml column buffer, proteins were eluted with 10 ml column buffer/10 mM maltose. Proteins were concentrated using Amicon ultra 50 kDa columns (Millipore, Watford, UK), and then confirmed by Coomassie staining and western blotting with anti-MBP (NEB). Protein concentrations were determined by Pierce BCA protein assay kit (Rockford, USA) according to the manufacturer's protocol.

### Analysis of MBP-fusion protein binding to HEp-2 cells by fluorescence microscopy

Binding of MBP-tagged Ifp was determined by fluorescence microscopy as described previously [18]. HEp-2 cells were cultured overnight on glass coverslips in 24-well plates at 2.5 × 10^5 ^cells/well in 1 ml tissue culture media (minimum essential medium eagle (MEM), 10% fetal calf serum (w/v), 2 mM L-glutamine, 100 U penicillin ml^-1^, 100 μg streptomycin ml^-1^, MEM non-essential amino acids). The cells were blocked for 30 minutes at 37°C/5% CO_2 _in 250 μl binding solution (0.4% BSA (w/v), 2.5 mM maltose, 2 mM L-glutamine in RPMI media), then incubated for 45 minutes at 37°C/5% CO_2 _with 250 μl MBP-Ifp, MBP-Ifp_C337G _or MBP protein alone (NEB) at 100 μg ml^-1 ^in binding solution. The cells were washed 5 times with 1 ml PBS/1% BSA (w/v) and incubated for 30 minutes at 37°C/5% CO_2 _in 250 μl of a 1:1000 dilution of rabbit anti-MBP antibody (NEB) in binding solution used. Cells were washed 5 times with 1 ml PBS/1% BSA (w/v) and incubated for 30 minutes at 37°C/5% CO_2 _in 250 μl of a 1:1000 dilution of goat anti-rabbit IgG Alexafluor 488 (Invitrogen) in binding solution. Cells were washed 4 times with 1 ml PBS/1% BSA (w/v) and fixed for 15 minutes at -20°C in 250 μl of 95% ethanol-5% acetic acid (v/v). The cover slips were removed from the wells, washed in Milli Q H_2_O and mounted onto glass slides with Vectashield-DAPI (Vector Laboratories, Peterborough, UK) mounting medium. The coverslips were examined using an Axiovert 200M (Zeiss, Welwyn Garden City, UK) confocal microscope. Experiment was performed on three independent occasions and at least 50 cells were examined per experiment.

### FACScan analysis of MBP-fusion protein binding to HEp-2 cells

A similar methodology was used as for the fluorescence microscopy as described previously [18], with the following modifications. The cells were grown directly in 6-well plates at 7 × 10^5 ^cells/well. The Alexafluor 488 anti-rabbit IgG antibody was diluted to 1:5000 in PBS/1% BSA (w/v). Cells were resuspended in PBS/0.5% EDTA (w/v) and transferred to BD Falcon 5 ml tubes (VWR, Lutterworth, UK). Cells were washed once with PBS/1% BSA (w/v) and centrifuged, then were fixed for 5 minutes on ice in 2% paraformaldehyde/PBS (w/v). The cells were washed once with PBS/1% BSA (w/v), centrifuged and then were resuspended in 500 μl PBS/1% BSA/0.02% EDTA (w/v). The fluorescence was measured using a FACScan machine (Becton Dickinson, Oxford, UK). Experiment was performed on two independent occasions and 20,000 cells were examined for fluorescence from each sample.

### Analysis of co-localisation of MBP-fusion protein and the receptors CD59 and β1 integrin on HEp-2 cells by fluorescence microscopy

A similar methodology was used as for the fluorescence microscopy described above with the following modifications. After the MBP-fusion protein incubation the cells were washed 5 times with PBS then incubated with 250 μl of a 1:20 dilution rabbit anti-Ifp (CovalAb, this study) and 1:1000 dilution of mouse anti-CD59 (Invitrogen) or a 1:1000 dilution of mouse anti-β1 integrin in binding solution for 30 minutes at 37°C/5% CO_2_. The cells were washed 5 times with 1 ml PBS then incubated with 250 μl of a 1:1000 dilution of goat anti-rabbit IgG Alexafluor 594 (Invitrogen) and 1:1000 dilution of goat anti-mouse IgG Alexafluor 488 (Invitrogen) for 30 minutes at 37°C/5% CO_2_. The cells were washed 5 times with 1 ml PBS then fixed for 30 minutes at 4°C with 250 μl 2% paraformaldehyde (w/v). The coverslips were removed from the wells, washed with PBS then mounted onto glass slides with Vectashield-DAPI mounting medium (Vector Laboratories). The slides were examined using an Axiovert 200 M confocal microscope (Zeiss). At least three areas of approximately 10 cells each were examined per sample and the experiment was performed on three independent occasions.

### Construction of *ifp *and *inv *insertional mutants

An *ifp *knockout mutant was generated in the *Y. pseudotuberculosis *strain IP32953, after initially constructing an ifp mutant in strain YPIII. Briefly, 1725 bp of *ifp *was amplified with IntA and IntB primers, digested with *Sac*I and *Sph*I then ligated into the cloning vector pGEM-T easy. The plasmid was digested with *Bgl*II to linearise and allow for the ligation of the kanamycin cassette within the *ifp *sequence. PCR with primers IntA and IntB was undertaken on the plasmid to create linear fragments of kanamycin cassette flanked by *ifp *sequence. This PCR product was electroporated into YPIII previously transformed with pKOBEG, which contains the λ red recombinase operon. The temperature sensitive pKOBEG plasmid was then lost from putative mutants by growth at 37°C, whilst the presence of the pYV plasmid was maintained by the addition of 2.5 mM CaCl2. Southern blot analysis confirmed correct mutation. Genomic DNA from this YPIIIΔ*ifp *was used as a template for PCR amplification of the kanamycin cassette flanked by two ~500 bp regions of gene-specific DNA. The primers INTA and INTB (Table 2) were used to amplify a 2.7 kbp product. This was purified using a Qiagen PCR purification kit, precipitated, and then resuspended in 5 μl MilliQ H_2_O. Strain IP32953 containing the mutagenesis plasmid pAJD434 [33] was grown in LB broth containing 100 μg trimethoprim ml^-1 ^and 0.8% arabinose (w/v) for 5 hours at 28°C in order to induce the expression of the λ-red genes from the pAJD434 plasmid. These cells were electroporated with the purified PCR product and kanamycin resistant colonies were screened by PCR and Southern blot to confirm the correct insertion. The pAJD434 plasmid was then removed by incubation overnight at 37°C in the presence of 2.5 mM CaCl_2_. Colonies were screened to confirm the loss of the pAJD434 plasmid and the presence of the virulence plasmid (pYV). A similar method was used for the construction of the *inv *mutant except primers YPTB1668Chlor1 and YPTB1668Chlor4 (Table 2), were designed to amplify the chloramphenicol resistant cassette from pBAD33 flanked by 50 bp gene-specific regions. This PCR product was then used as described above to generate an insertional mutant of the *inv *gene (IPΔINV) and a double *ifp *and *inv *insertional mutant (IPΔIFPΔINV), by electroporation into IP32953 WT or mutated *ifp *(IPΔIFP) strains. Colonies were screened as above with *inv *specific primers and the presence of pYV and loss of pAJD434 was confirmed.

### Construction of *ifp *complement in pBAD33 plasmid

The *ifp *gene including native promoter was amplified by PCR using specific primers INTPROM3 + INTPROM4 (Table 2). After ligation into pGEM-T Easy vector (Promega) the construct was transformed into XL2-Blue *E. coli *(Stratagene, La Jolla, USA). The construct was screened by PCR and sequenced, before the *ifp *gene with promoter was digested from the pGEM-T Easy vector with *Kpn*I and *Sph*I and purified by gel extraction using a Gen Elute purification kit (Sigma). This insert was cloned into a pBAD33 plasmid [34], also digested with *Kpn*I and *Sph*I and transformed into TOP10 *E. coli *(Invitrogen). These colonies were again screened by PCR and by digestion with *Eco*RV to confirm the correct insert and orientation within the pBAD33 vector. IPΔIFP cells were made competent by washing 3 times in 10 ml ice cold H_2_O and electroporated with pBAD33*ifp *(pIFP) plasmid to generate an *ifp *mutant with a complemented *ifp *gene (IPΔIFPpIFP). These were screened by PCR and were DNA sequenced again to confirm the presence of the correct complemented gene.

### Plasmid cured strains

Wild type, defined mutants and *ifp *complemented mutant strains lacking the pYV plasmid were generated by culturing strains overnight at 37°C the selecting for white colonies on CRMOX plates [31]. Loss of pYV was verified by PCR and repeated screening on CRMOX.

### Adhesion and invasion of HEp-2 cells

HEp-2 cells were cultured overnight at 37°C 5% CO_2 _on coverslips in 24-well plates at 2 × 10^5 ^cells/well in 1 ml tissue culture medium. The 10 ml LB broth cultures of IP32953 wild type (IPWT), defined mutants (IPΔIFP, IPΔINV, IPΔIFPΔINV) and mutant with complemented *ifp *(IPΔIFPpIFP), were incubated at 37°C for 14 hours with appropriate antibiotics and 2.5 mM CaCl_2_. The cells were washed 3 times with 1 ml PBS and then, at a multiplicity of infection (MOI) of 70:1, incubated for 1 hour with 1 ml of bacterial culture in MEM media at 37°C, 5% CO_2_. Inoculum was plated on LB agar to determine number of colony forming units (cfu). The cells were washed 5 times with 1 ml PBS and then fixed with 2% paraformaldehyde (w/v) for 45 minutes at 4°C, before being washed again 5 times with 1 ml PBS. The coverslips were incubated with a 1:500 dilution of anti-*Yersinia pseudotuberculosis *antibody (Abcam, Cambridge, UK) in PBS for 45 minutes at room temperature. The coverslips were washed with PBS then incubated with a 1:1000 dilution of anti-rabbit IgG Alexafluor 488 (green) (Invitrogen) in PBS for 45 minutes at room temperature. After washing with PBS the cells were permeabilised with 0.1% Triton X100-PBS (v/v) for 20 minutes at room temperature. The coverslips were washed with PBS and incubated with 1:500 dilution of anti-*Y. pseudotuberculosis *antibody (Abcam) in PBS for 45 minutes at room temperature, before being washed again with PBS. Anti-rabbit IgG Alexafluor 594 (red) (Invitrogen) was diluted 1:1000 in PBS and the coverslips were incubated for 45 minutes at room temperature. The coverslips were washed with PBS and mounted onto slides with Vectorshield-DAPI mounting media (Vector Laboratories). Slides were examined by Axiovert 200 M (Zeiss) confocal microscope. Three coverslips per strain with 100 HEp-2 cells per coverslip were counted, utilising the z stack images to gain a 3D representation of the cell. Adhesion and invasion were quantified by counting invaded (red) and adhered (red/green) bacteria and calculating percentage adhesion or invasion per HEp-2 cell based on known MOI. This adhesion and invasion assay was performed on at least three independent occasions.

### Detection of the presence of invasin by western blot

Strains were cultured for 15 hours at 28°C and 37°C, the OD_600 nm _measured and cultures adjusted so all strains had equal quantities of bacteria/ml. Strains were run on a SDS-PAGE 12% Bis-Tris gel, blotted onto nitrocellulose membrane and blocked with 5% milk-PBS-0.1% Tween20. Invasin was visualised by staining with anti-invasin monoclonal antibody [35] at 1:10000 and anti-rabbit IgG peroxidase conjugate (Sigma) secondary antibody at 1:10000.

### *Galleria mellonella *model of infection

*G. mellonella *larvae were purchased from Livefood UK Ltd (Rooks Bridge, Somerset, UK). Larvae were infected with 10^6 ^cfu *Y. pseudotuberculosis *IP32953WT, IPΔIFP, IPΔINV, IPΔIFPΔINV or IPΔIFPpIFP in 10 μl inocula by micro-injection (25 μl Hamilton syringe, Cole Palmer, London, UK) in the right foremost leg. PBS and no injection controls were used. The larvae were incubated at 37°C and survival at 72 hours post-infection was recorded. Larvae were scored as dead when the colouration changed from normal pale cream to brown and failed to respond even after gentle manipulation with a pipette tip.

## Results

### Identification of an intimin and invasin-like protein in *Y. pseudotuberculosis*

**Figure 1:**
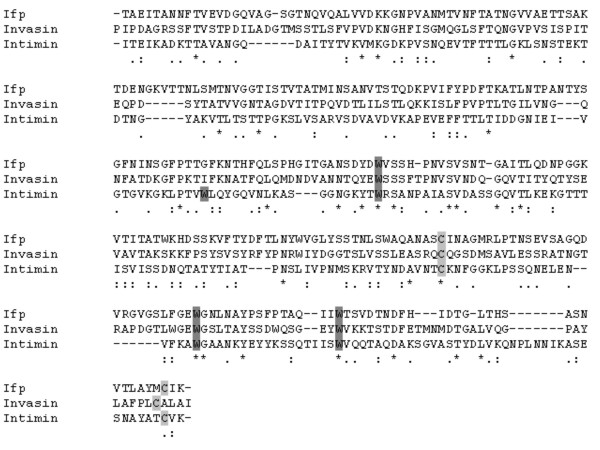
**Amino acid alignment of intimin, ifp and invasion**. The C-terminal 280 residues of EPEC 2348/69 intimin (Int280) and the corresponding regions from *Y. pseudotuberculosis *IP32953 Ifp and invasin were aligned. Residues important in intimin function are shown: conserved cysteine residues are highlighted in light grey whilst tryptophan residues are highlighted in dark grey.

The genome sequence of *Y. pestis *strain CO92 first revealed the potential presence of an intimin-like protein [36]. However, in this sequence and all other subsequently sequenced *Y. pestis *strains, the predicted coding sequence for the intimin-like protein is disrupted by an IS285 element, or in the instance of strain 91001, a premature stop codon. By contrast, this gene is intact in all four *Y. pseudotuberculosis *strains sequenced to date, with at maximum, only six amino acid differences between these strains (Additional file 1). Alignments with the European Bioinformatics Institute (EBI) EMBOSS Pairwise Alignment tool [37] revealed that the translated full-length coding sequence of IP32953 Ifp has 33.9% amino acid identity (or 46.7% similarity) to *Y. pseudotuberculosis *IP32953 invasin, and 29.7% amino acid identity (42.8% similarity) to the α-subtype of intimin (*eaeA*) from E2348/69 *E. coli*, therefore this gene was termed *ifp*. Using the ClustalW program (SDSC Workbench, San Diego, USA) alignment of the C terminal domains of invasin, intimin and Ifp, reveals the presence of two cysteine residues in Ifp, which are important for the function of invasin and intimin (Figure 1). In addition three of the four tryptophans, which are present in all intimin subtypes [27], are also present in Ifp and invasin (Figure 1).

### Thermoregulated temporal expression of *ifp *and *inv*

**Figure 2:**
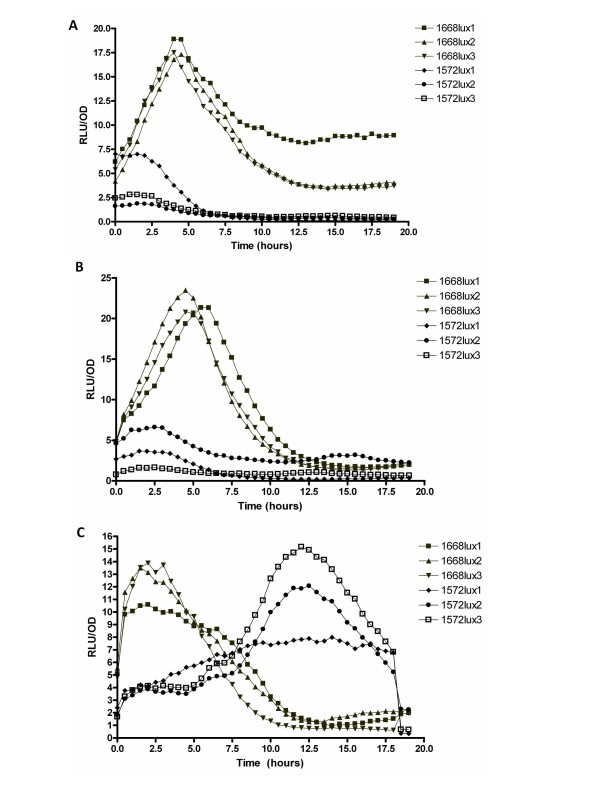
**Temporal expression of *ifp *(*1572lux*) and *inv *(*1668lux*)**. Expression was determined by light emission from *lux*-reporter strains grown at (A) 24°C, (B) 28°C and (C) 37°C. Three biological replicates are shown for each strain, with each biological replicate tested in triplicate.

The expression of *ifp *(*YPTB1572*) and *inv *(*YPTB1668*) at 24°C, 28°C and 37°C, were monitored using *lux*-based promoter fusions *1572lux *and *1668lux *in *Y. pseudotuberculosis *IP32953, with the resultant luminescence read in a Lucy1 combined photometer and luminometer (Figure 2). The expression was determined as relative light units/optical density (RLU/OD) therefore the growth phase could also be determined, based on these OD readings (Additional file 2). *Inv *was maximally expressed during log phase after 5 hours at 24°C and 28°C, but after only 2.5 hours at 37°C, suggesting that mammalian body temperature is important in the induction of *inv *and confirms the observation of Isberg *et al. *[38]. In contrast, *ifp *expression remains low at 24°C and 28°C throughout the time course, whereas at 37°C there was little expression in first 7.5 hours, after which expression increases to a peak at 13 hours (Figure 2).

### Ifp binds to localised foci on HEp-2 cells

**Figure 3:**
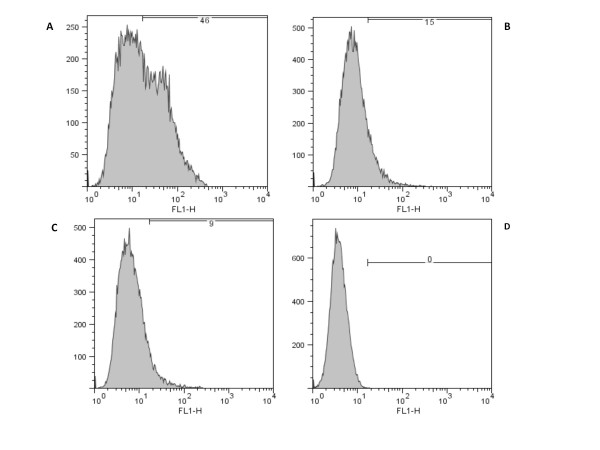
**FACScan analysis of the binding of purified MBP-fusion proteins to HEp-2 cells**. Cells were incubated with (A) MBP-Ifp, (B) MBP-Ifp_C337G_, (C) MBP or (D) PBS and binding was visualised with anti-MBP and anti-rabbit Alexafluor 488 antibodies.

**Figure 4:**
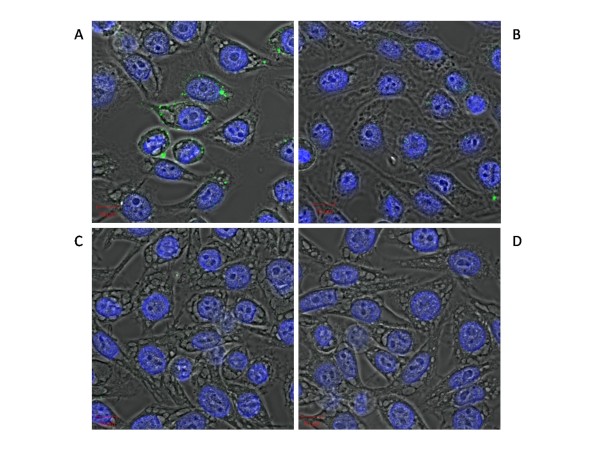
**Binding of purified MBP-fusion proteins to HEp-2 cells**. Cells were incubated with (A) MBP-Ifp, (B) MBP-Ifp_C337G_, (C) MBP or (D) PBS and binding was visualised with anti-MBP and anti-rabbit Alexafluor 488 antibodies. Representative cells are shown and the 10 μm ruler is shown in red.

Invasin and intimin are able to bind directly to specific receptors on the surface of mammalian cells. We therefore investigated the ability of Ifp to bind directly to HEp-2 cells using a MBP tagged Ifp purified protein (MBP-Ifp). In addition, to determine if the terminal cysteine was as important in Ifp functionality (as it is in invasin and intimin), a MBP-Ifp recombinant protein with the terminal cysteine mutated to a glycine (MBP-Ifp_C337G_) was constructed and tested. Utilising flow cytometry, FACScan analysis showed a shift in the peak of fluorescence of HEp-2 cells which had been incubated with MBP-Ifp (Figure 3). This shift was not seen with cells incubated with MBP-Ifp_C337G _or MBP alone, indicating not only that this is a specific binding of MBP-Ifp, but also that the terminal cysteine is important in the functional binding of Ifp to HEp-2 cells. However, MBP-Ifp only appears to bind to a subset of cells and to differing levels, as shown by the width of the shifted peak. In order to investigate this further, we used confocal microscopy to visualise the binding of MBP-Ifp to HEp-2 cells (Figure 4). Again no specific binding is seen with the MBP control and binding is greatly reduced with MBP-Ifp_C337G_, whilst the MBP-Ifp fusion protein binds to individual cells with significant levels of fluorescence visible. Of 50 cells examined ~40% showed MBP-Ifp adherence, with only ~15% showing MBP-Ifp_C337G _adhesion. Of those showing MBP-Ifp_C337G _adherence, fewer fluorescing spots were observed per cell compared to MBP-Ifp, and these spots were smaller.

**Figure 5:**
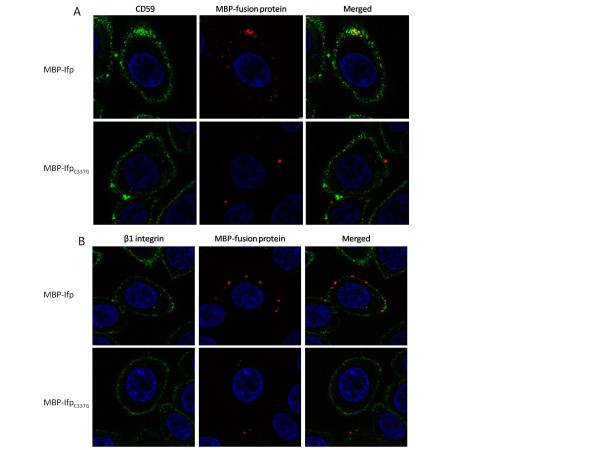
**Fluorescence microscopy showing co-localisation of (A) CD59 and (B) β1 integrin with purified MBP-fusion proteins on HEp-2 cells**. Cells were incubated with MBP-Ifp or MBP-Ifp_C337G_. MBP-fusion proteins were visualised with anti-Ifp and anti-rabbit Alexafluor 594 antibodies. CD59 was visualised with anti-CD59 and anti-mouse Alexafluor 488 antibodies. β1 integrin was visualised with anti-β1 integrin and anti-mouse Alexafluor 488 antibodies. Representative cells are shown.

Interestingly, this binding appears to be localised to specific foci on the cell surface, rather than a random scattering of fluorescence across the entire cell surface. This suggests that the protein is binding to specific receptors on the cell surface which are localised in foci. In order to investigate if a putative receptor was localised in cholesterol and sphingolipid-enriched plasma membrane micro-domains (lipid rafts), we used co-localisation assays. In this instance the GPI-anchored protein CD59, which is known to localise to these microdomains [39], was used as a marker for the position of the lipid rafts. Confocal microscopy revealed that there is co-localisation between CD59 and MBP-Ifp bound on the cell surface, indicating that there is a putative receptor for Ifp present within these lipid rafts (Figure 5A). However, as there is binding of MBP-Ifp which does not co-localise, and as invasin is known to bind to β1 integrin, co-localisation between MBP-Ifp and β1 integrin was also investigated (Figure 5B). No co-localisation was observed between MBP-Ifp and β1 integrin.

### Adhesion and invasion assays

**Figure 6:**
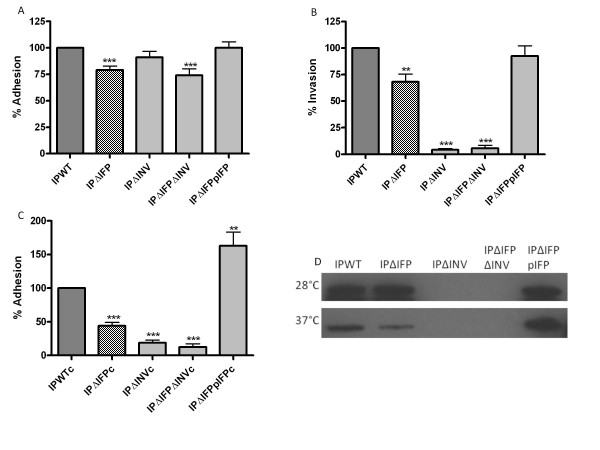
**Adhesion and invasion of HEp-2 cells by wild type IP32953 and defined mutants**. (A) adhesion, (B) invasion (C) adhesion with pYV cured strains, using differential staining assay. Wild type (IPWT) was compared to insertional mutants of *ifp *(IPΔIFP) and *inv *(IPΔINV), an *ifp *and *inv *double mutant (IPΔIFPΔINV) and an *ifp *mutant with complemented *ifp *(IPΔIFPpIFP), by setting IPWT values to 100%. Strains cured of pYV are marked with "c". Data was pooled from assays performed in triplicate on at least three independent occasions with statistical analysis by unpaired t-test and statistically significant results designated by *. ** indicates p value of <0.005, *** indicates p value of <0.0005. (D) Presence of invasin at 28°C and 37°C after 15 hours incubation detected by western blotting with anti-invasin monoclonal antibody.

In order to confirm the role of Ifp as an adhesin, we constructed an insertion mutant in the *ifp *coding sequence of *Y. pseudotuberculosis *strain IP32953 (IPΔIFP). For comparative purposes, we also constructed an insertion mutant in the *inv *gene (IPΔINV), and a double insertion mutant (IPΔIFPΔINV) in the same strain. To determine the level of adhesion and invasion of these defined mutants, in comparison to wild type, a differential staining assay [40] was utilised. Figure 6 shows that there is a significant decrease in the level of adhesion of IPΔIFP compared to wild type (IPWT), which could be restored by complementation with the wild type *ifp *gene (IPΔIFPpIFP) (Figure 6A). The *inv *mutant did not show as great a reduction in adhesion as IPΔIFP, but the double mutant showed comparable levels to the *ifp *single mutant. A significant decrease in invasion of IPΔIFP compared to wild type is observed (Figure 6B). Both IPΔINV and IPΔIFPΔINV show significant decreases in invasion compared to wild type; however, it was beyond the sensitivity of this assay to determine slight differences between these two strains. The average ratio of intracellular:extracellular bacteria for each of the strains associated with the HEp-2 cells was as follows; IPWT 1:8; IPΔIFP 1:8; IPΔINV 1:176; IPΔIFPΔINV 1:141 and IPΔIFPpIFP 1:8. To determine the role of the virulence factors of the pYV in the adhesion and invasion still seen in these assays, the strains were cured of the pYV plasmid and the differential staining assay repeated (Figure 6C). Invasion levels were all below the sensitivity of this assay, but a significant difference was observed between wild type and IPΔIFP, IPΔINV and IPΔIFPΔINV for adhesion. Although the expression analysis suggested the invasin should not be expressed at the time point used for these experiments, as there was a significant difference between wild type and *inv *mutants, presence of invasin was examined by western blot (Figure 6D). Invasin was found to still be present at 37°C although at a reduced level compared to 28°C 15 hour cultures. No invasin was observed in IPΔINV and IPΔIFPΔINV which confirms the mutation of the *inv *gene in these strains.

### *Galleria *model of infection

**Figure 7:**
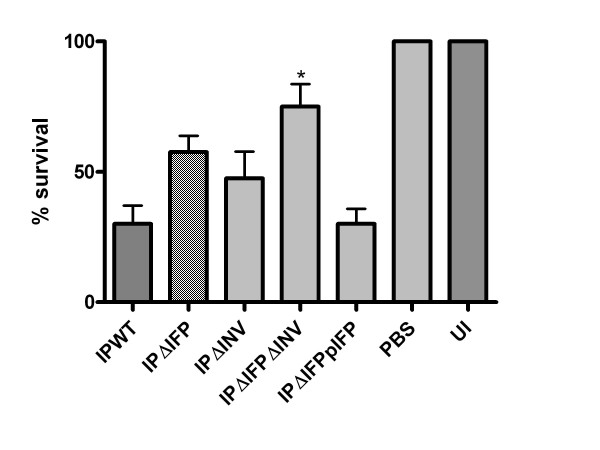
**Survival of *G. mellonella *following infection with 10^6 ^cfu per larva of *Y. pseudotuberculosis *wild type IP32953 and defined mutants**. Wild type (IPWT) was compared to insertional mutants of *ifp *(IPΔIFP) and *inv *(IPΔINV), an *ifp *and *inv *double mutant (IPΔIFPΔINV) and an *ifp *mutant with complemented *ifp *(IPΔIFPpIFP). Phosphate buffered saline (PBS) injection and uninfected (UI) *Galleria *were utilised as controls. Assays were performed on at least three independent occasions, each with 10 larvae per strain. Statistical analysis by non-parametric t-test with statistically significant results marked with * (p =< 0.05).

*Galleria mellonella *has been used as an infection model for several bacterial pathogens because it possesses an innate immune system with structural and functional homology to the mammalian immune system. Consequently it can be utilised as a less labour intensive and more cost effective model, in comparison to the traditional murine model of *in vivo *infection [41]. This model has been used recently for infection studies with *Y. pseudotuberculosis *[42]. Therefore, in addition to the adhesion and invasion assays, the ability of the mutants to infect and kill the wax moth larvae *G. mellonella *was examined. Bacteria, which had been cultured overnight at 37°C, were injected into the foreleg of the *G. mellonella *at 10^6 ^colony forming units (cfu) per 10 μl injection. After 72 hours at 37°C the number of dead *G. mellonella *were enumerated (Figure 7); larvae were scored as dead if they had become melanised and ceased moving [42]. Both IPΔIFP (average 58% survival, p = 0.057) and IPΔINV (average 48% survival, p = 0.200) mutants showed modest if not significant attenuation in the *G. mellonella *model, compared to wild type IP32953 (average 30% survival). IPΔIFPpIFP shows similar levels of virulence to IPWT (average 30% survival, p = 0.857). Average survival of 75% was recorded in larvae infected with the double mutant, which showed a significant difference to the wild type (p = 0.028) when analysed by non-parametric t-test (Graphpad Prism 4, La Jolla, USA).

## Discussion

In this study we investigated the role of a novel *Y. pseudotuberculosis *adhesin (Ifp), which shows similarity to both invasin and the intimin adhesin of EPEC and EHEC (Figure 1). As invasin and intimin are well characterised virulence determinants, the discovery of a new member in the same family of outer membrane adhesins, is intriguing. The predicted coding sequence for *ifp *is disrupted in all seven currently sequenced strains of *Y. pestis*, although it is intact in the four *Y. pseudotuberculosis *strains sequenced. This disruption is due to an insertion element (IS285) in all *Y. pestis *strains, with the exception of the atypical 91001 *Y. pestis Microtus *strain, where it is disrupted by a nonsense mutation [3,43,44]. This may suggest that the disruption in this gene in *Y. pestis *occurred early in the divergence of *Y. pestis *from *Y. pseudotuberculosis *and may have been a potentially important step in this evolutionary process. The *inv *gene of *Y. pseudotuberculosis *is also disrupted by an insertion element (IS200) in *Y. pestis *[45]. The reason for the loss of function of invasin and Ifp in *Y. pestis *is unknown, but given that it does not have an enteric phase in its lifecycle, it is likely to be not required. This follows the "use it or lose it" concept, whereby genes which no longer provide a selective advantage to the bacteria become pseudogenes [46]. The amino acid similarity of Ifp to both invasin and intimin, coupled with its retention in *Y. pseudotuberculosis*, suggests a putative role for Ifp in adhesion to cells and that Ifp is a new member in the family of surface adhesins together with invasin and intimin.

The C-terminal 280 amino acids of intimin are the functional domain in this adhesin [28], and two cysteine (C859 and C937; numbering according to EPEC strain E2348/69) and four tryptophan (W776, W795, W881 and W899) residues are conserved between all intimin subtypes. An alignment of the C-terminal region amino acid sequences of α-subtype intimin from EPEC, *Y. pseudotuberculosis *invasin and Ifp, revealed that both cysteine and three out of the four tryptophan residues were found to be conserved in both Ifp and invasin (Figure 1). Only W776 was not conserved in Ifp and invasin. These cysteine and tryptophan residues are involved in receptor binding by intimin [27,30] and therefore may have a role in Ifp receptor binding.

We demonstrated direct binding of Ifp using both flow cytometry and fluorescence microscopy, where the MBP-Ifp fusion proteins could bind to HEp-2 cells. By contrast the MBP alone did not bind and the specific cysteine residue mutant MBP-Ifp_C337G _showed greatly reduced levels of binding (Figures 3 and 4). This reduced level of binding with the MBP-Ifp_C337G _shows that the cysteine residue is important to allow Ifp binding. The same cysteine residue is known to be important in both intimin and invasin, through the formation of a disulphide bond [18,29], therefore it may be that the cysteine has a similar role in Ifp. The width of the FACS fluorescence intensity graph suggests that MBP-Ifp does not bind uniformly to all cells (Figure 3). This was confirmed by confocal microscopy, where the cells which were exposed to the MBP-Ifp fusion protein, showed a pattern of fluorescence of intensely stained localised areas, instead of scattered fluorescence across the cell surface. A similar pattern of adherence was observed when HEp-2 cells were incubated with MBP fusion proteins of intimin and invasin [18]. As invasin is known to bind to β1 integrins and it has been suggested that intimin can bind to β1 integrin [13,24,25] co-localisation of Ifp to β1 integrin was investigated (Figure 5B). As no co-localisation was observed it shows that Ifp binds to alternative receptors on the cell surface. Lipid rafts are sphingolipid and cholesterol rich regions of the plasma membrane, into which Tir is thought to be transferred [47,48]. Additionally uropathogenic *E. coli *are known to invade via lipid rafts [49], and *Salmonella *and *Shigella sp. *use a type three secretion system to translocate effectors, by binding to cholesterol within lipid rafts [50]. To investigate if MBP-Ifp was binding to a component of these lipid rafts, co-localisation of MBP-Ifp to CD59 was undertaken by confocal microscopy. CD59 was selected as it is known to localise to these micro-domains and could therefore act as a marker. The results show co-localisation of Ifp and CD59, which was reduced with MBP-Ifp_C337G _(Figure 5A), suggesting that there is a putative receptor for Ifp within these lipid rafts. The Ifp receptor within these lipid rafts has yet to be determined, but as not all of the MBP-Ifp co-localised, no conclusions can currently be made as to the exact receptor of Ifp.

*Inv *is differentially thermoregulated with lower levels being expressed at 37°C compared to 28°C [38]. In comparison, *yadA *shows maximal expression at 37°C in exponential phase culture, conditions where *inv *expression is repressed [51]. YadA is a virulence plasmid (pYV) encoded adhesin, known to be involved during the infection of *Y. pseudotuberculosis *[51-53]. The pattern of *inv *expression was confirmed by this study, where *inv *was expressed both at 28°C and 37°C during lag and early log phase culture, although at a greater degree at 28°C (Figure 2). The *ifp *gene appears to be expressed at 37°C at a later time point in the late log or early stationary phase, when *inv *expression is reduced. As *ifp *and *yadA *are expressed at similar time points and at the same temperature, Ifp may have a similar role to YadA during the infection of *Y. pseudotuberculosis *[51]. Although *inv *expression is decreased at a later time point, it still appears to have an effect on the invasion of *Y. pseudotuberculosis *(Figure 6B); this is despite using stationary phase cultures which had been grown at 37°C. The western blot analysis for presence of invasin under these conditions (Figure 6D), confirmed that although *inv *may no longer be actively expressed, invasin was still present in the cell and could therefore have a role in invasion of HEp-2 cells.

The invasion and adhesion assays confirmed the microscopy and flow cytometry results, in demonstrating a role for Ifp as an adhesin, as the levels of adhesion were reduced with IPΔIFP in comparison to wild type (Figure 6A). The *inv *mutant did not show as great a decrease in adhesion as the *ifp *mutant, but the double mutant showed similar if not a marginally greater reduction in adhesion as IPΔIFP, in comparison to the wild type. Although levels of invasion were significantly affected by IPΔIFP, this may be due to reduced adhesion, suggesting that Ifp is an adhesin. Any differences between IPΔINV and IPΔIFPΔINV were beyond the detection capability of this assay, but it appeared that invasin was the dominant protein involved in the invasion of the HEp-2 cells. Removal of the pYV and therefore the YadA and Yop virulence factors allowed greater distinction of the role of Ifp. Without these extra virulence determinants compensating for the mutation of *ifp*, the IPΔIFP mutant showed a statistically significant reduction in adhesion compared to IPWT (Figure 6C). This confirms the role of Ifp as an adhesin of *Y. pseudotuberculosis*.

As *G. mellonella *possesses an innate immune system with structural and functional similarities to the mammalian innate immune system, it is a useful alternative to the traditional murine yersiniosis infection model, to examine virulence *in vivo*, especially as unlike the *C. elegans *model, *G. mellonella *can be incubated at 37°C [42,54]. Previous studies with *Y. pseudotuberculosis *comparing *G. mellonella *and the murine model, showed that *G. mellonella *could reflect infection in mammals and therefore could be useful as a higher throughput screen of mutants, before a more in depth analysis was undertaken in the murine model [42]. In this study the *G. mellonella *model demonstrated a role for Ifp in the pathogenesis of *Y. pseudotuberculosis*, in particular in concert with invasin, as the double mutant showed a significant increase in survival compared to the wild type (Figure 7). There also appeared to be mild attenuation in virulence with both of the single mutants. This suggests that Ifp, together with invasin, does have a role in virulence of *Y. pseudotuberculosis *in this infection model.

## Conclusions

We have shown the presence of a novel functional adhesin in *Y. pseudotuberculosis *that has been mutated with an IS element and is presumably non-functional in *Y. pestis. Ifp *is expressed during late log to early stationary phase at 37°C and demonstrates an ability to bind to HEp-2 cells *in vitro*, which can be disrupted by mutation of the gene, or even a single cysteine residue. Together with invasin and intimin, Ifp is a new member of a family of outer membrane adhesins that is activated at 37°C and may act at a later stage than invasin during infection.

## Authors' contributions

All authors read and approved the manuscript and contributed to experimental design. PS and BW contributed to manuscript preparation.

## Supplementary Material

Additional file 1 **Amino acid alignment of Ifp from the four currently sequenced genomes of *Y. pseudotuberculosis***. Utilising the ClustalW program, the amino acid sequences of *Y. pseudotuberculosis *strains IP32953, IP31758, PB1 and YPIII were aligned. Click here for file

Additional file 2 **Growth curves from the temporal expression of *Ifp *and *invasin *assay**. Within the Anthos Lucy1 combined photometer and luminometer, OD readings at 600 nm were taken at 30 minute intervals and used to construct these growth curves. Cultures were incubated at (A) 24°C (B) 28°C and (C) 37°C. Click here for file
