# Supplementary material for: Identification and characterisation of a novel adhesin Ifp in Yersinia pseudotuberculosis
Source: BMC Microbiol. 2011 Apr 28;11:85. doi: 10.1186/1471-2180-11-85 (PMC3102037; doi:10.1186/1471-2180-11-85)
Supplement: Additional file 1 — Amino acid alignment of Ifp from the four currently sequenced genomes of Y. pseudotuberculosis. Utilising the ClustalW program, the amino acid sequences of Y. pseudotuberculosis strains IP32953, IP31758, PB1 and YPIII were aligned. [file 1471-2180-11-85-S1.DOC]

**Supplementary figure 1** Amino acid alignment of Ifp from the four currently sequenced genomes of *Y. pseudotuberculosis*. ClustalW program was used from SDSC Workbench website for this alignment.

PB1 MSLYRISSLHQAKQLNKNKQLNKTRISKSVVWANIVIQAIFPLSIAFTPAVMAAETVGAS

IP32953 MSLYRISSLHQAKQLNKNKQLNKTRISKSVVWANIVIQAIFPLSIAFTPAVMAAETVGAS

YPIII MSLYRISSLHQAKQLNKNKQLNKTRISKSVVWANIVIQAIFPLSIAFTPAVMAAETVGAS

IP31758 MSLYRISSLHQAKQLNKNKQLNKTRISKSVVWANIVIQAIFPLSIAFTPAVMAAETVGAS

************************************************************

PB1 DEKPRSASQAEQSTANAATRLASILTNDDSTKQASSIARGTAANAGNEALQKWFNQFGSA

IP32953 DEKPRSASQAEQSTANAATRLASILTNDDSTKQASSIARGTAANAGNEALQKWFNQFGSA

YPIII DEKPRSASQAEQSTANAATRLASILTNDDSAKQASSIARGTAANAGNEALQKWFNQFGSA

IP31758 DEKPRSASQAEQSTANAATRLASILTNDDSAKQASSIARGTAANAGNEALQKWFNQFGSA

******************************:*****************************

PB1 KVQLNLDEKLSLKGSQLDVLLPLTDSPDLLTFTQLGGRYIDDRVTLNVGLGQRHFFAQQM

IP32953 KVQLNLDEKLSLKGSQLDVLLPLTDSPDLLTFTQLGGRYIDDRVTLNVGLGQRHFFAQQM

YPIII KVQLNLDEKLSLKGSQLDVLLPLTDSPDLLTFTQLGGRYIDDRVTLNVGLGQRHFFAQQM

IP31758 KVQLNLDEKLSLKGSQLDVLLPLTDSPDLLTFTQLGGRYIDDRVTLNVGLGQRHFFAQQM

************************************************************

PB1 LGYNLFVDHDASYSHTRIGVGAEYGRDFINLAANGYVGVSGWKNSPDLDKYDEKVANGFD

IP32953 LGYNLFVDHDASYSHTRIGVGAEYGRDFINLAANGYVGVSGWKNSPDLDKYDEKVANGFD

YPIII LGYNLFVDHDASYSHTRIGVGAEYGRDFINLAANGYFGVSGWKNSPDLDKYDEKVANGFD

IP31758 LGYNLFVDHDASYSHTRIGVGAEYGRDFINLAANGYFGVSGWKNSPDLDKYDEKVANGFD

************************************.***********************

PB1 LRSEAYLPTLPQLGGKLIYEQYFGDEVGLFGVDNRQKNPLAVTLGVNYTPIPLFTVGVDH

IP32953 LRSEAYLPTLPQLGGKLIYEQYFGDEVGLFGVDNRQKNPLAVTLGVNYTPIPLFTVGVDH

YPIII LRSEAYLPTLPQLGGKLIYEQYFGDEVGLFGVDNRQKNPLAVTLGVNYTPIPLFTVGVDH

IP31758 LRSEAYLPTLPQLGGKLIYEQYFGDEVGLFGVDNRQKNPLAVTLGVNYTPIPLFTVGVDH

************************************************************

PB1 KMGRAGMNDTRFNLGFNYAFGTPLAHQLDSDAVAIKRSLMGSRYNLVDRNNQIVMKYRKQ

IP32953 KMGRAGMNDTRFNLGFNYAFGTPLAHQLDSDAVAIKRSLMGSRYNLVDRNNQIVMKYRKQ

YPIII KMGRAGMNDTRFNLGFNYAFGTPLAHQLDSDAVAIKRSLMGSRYNLVDRNNQIVMKYRKQ

IP31758 KMGRAGMNDTRFNLGFNYAFGTPLAHQLDSDAVAIKRSLMGSRYNLVDRNNQIVMKYRKQ

************************************************************

PB1 NRVTLELPARVSGAARQTMPLVANATAQQGIDRIEWEASALTLAGGKITGSGNNWQITLP

IP32953 NRVTLELPARVSGAARQTMPLVANATAQQGIDRIEWEASALTLAGGKITGSGNNWQITLP

YPIII NRVTLELPARVSGAARQTMPLVANATAQQGIDRIEWEASALTLAGGKITGSGNNWQITLP

IP31758 NRVTLELPARVSGAARQTMPLVANATAQQGIDRLEWEASALTLAGGKITGSGNNWQITLP

*********************************:**************************

PB1 SYLSGGEGNNTYRISAIAYDTLGNASPVAYSDLVVDSHGVNTNASGLTAAPEILPANASA

IP32953 SYLSGGEGNNTYRISAIAYDTLGNASPVAYSDLVVDSHGVNTNASGLTAAPEILPANASA

YPIII SYLSGGEGNNTYRISAIAYDTLGNASPVAYSDLVVDSHGVNTNASGLTAAPEILPANASA

IP31758 SYLSGGEGNNTYRISAIAYDTLGNASPVAYSDLVVDSHGVNTNASGLTAAPEILPANASA

************************************************************

PB1 SSVIEFNIKDNANQPITGIADELAFSLELVELPEELAKAKARSVPLKTVSHTLTKITESA

IP32953 SSVIEFNIKDNANQPITGIADELAFSLELVELPEELAKAKARSVPLKTVSHTLTKITESA

YPIII SSVIEFNIKDNANQPITGIADELAFSLELVELPEELAKAKARSVPLKTVSHTLTKITESA

IP31758 SSVIEFNIKDNANQPITGIADELAFSLELVELPEELAKAKARSVPLKTVSHTLTKITESA

************************************************************

PB1 PGIYQATLTSGSKPQLINITAQINGVPLADVQTKVTLIADESTATLQTSSLQIITNGSLA

IP32953 PGIYQATLTSGSKPQLINITAQINGVPLADVQTKVTLIADESTATLQTSSLQIITNGSLA

YPIII PGIYQATLTSGSKPQLINITAQINGVPLADVQTKVTLIADENTATLQTSSLQIITNGSLA

IP31758 PGIYQATLTSGSKPQLINITAQINGVPLADVQTKVTLIADQSTATLQTSSLQIITNGSLA

****************************************:.******************

PB1 DDTDANQIRAVVVDAYGNKLSGVQVNFTVGNNAKITETTLSDKQGGVTAAITSTKAGTYT

IP32953 DDTDANQIRAVVVDAYGNKLSGVQVNFTVGNNAKITETTLSDKQGGVTAAITSTKAGTYT

YPIII DDTDANQIRAVVVDAYGNKLSGVQVNFTVGNNAKITETTLSDKQGGVTAAITSTKAGTYT

IP31758 DDTDANQIRAVVVDAYGNKLSGVQVNFTVGNNAKITETTLSDKQGGVTAAITSTKAGTYT

************************************************************

PB1 VTAELNGVTQQIDVNFIPDAGTATLDDSDEYKLQWVTNGQVADGESTNSVQLTVVDKFGN

IP32953 VTAELNGVTQQIDVNFIPDAGTATLDDSDEYKLQWVTNGQVADGESTNSVQLTVVDKFGN

YPIII VTAELNGVTQQIDVNFIPDAGTATLDDSDEYKLQWVTNGQVADGESTNSVQLTVVDKFGN

IP31758 VTAELNGVTQQIDVNFIPDAGTATLDDSDEYKLQWVTNGQVADGESTNSVQLTVVDKFGN

************************************************************

PB1 TVPGVDVAFTTDIGAIISEVTPTDANGVATAKIISSQAKSHTVKATLNRKEQTVEVNFIA

IP32953 TVPGVDVAFTTDIGAIISEVTPTDANGVATAKIISSQAKSHTVKATLNRKEQTVEVNFIA

YPIII TVPGVDVAFTTDIGAIISEVTPTDANGVATAKIISSQAKSHTVKATLNRKEQTVEVNFIA

IP31758 TVPGVDVAFTTDIGAIISEVTPTDANGVATAKIISSQAKSHTVKATLNRKEQTVEVNFIA

************************************************************

PB1 DTATAEITANNFTVEVDGQVAGSGTNQVQALVVDKKGNPVANMTVNFTATNGVVAETTSA

IP32953 DTATAEITANNFTVEVDGQVAGSGTNQVQALVVDKKGNPVANMTVNFTATNGVVAETTSA

YPIII DTATAEITANNFTVEVDGQVAGSGTNQVQALVVDKKGNPVANMTVNFTATNGVVAETTSA

IP31758 DTATAEITANNFTVEVDGQVAGSGTNQVQALVVDKKGNPVANMTVNFTATNGVVVETTSA

******************************************************.*****

PB1 KTDENGKVTTNLSMTNVGGTISTVTATMINSANVTSTQDKPVIFYPDFTKATLNTPANTY

IP32953 KTDENGKVTTNLSMTNVGGTISTVTATMINSANVTSTQDKPVIFYPDFTKATLNTPANTY

YPIII KTDENGKVTTNLSMTNVGGTISTVTATMINSANVTSTQDKPVIFYPDFTKATLNTPANTY

IP31758 KTDENGKVTTNLSMTNVGGTISTVTATMINSANVTSTQDKPVIFYPDFTKATLNTPANTY

************************************************************

PB1 SGFNINSGFPTTGFKNTHFQLSPHGITGANSDYDWVSSHPNVSVSNTGAITLQDNPGGKV

IP32953 SGFNINSGFPTTGFKNTHFQLSPHGITGANSDYDWVSSHPNVSVSNTGAITLQDNPGGKV

YPIII SGFNINSGFPTTGFKNTHFQLSPHGITGANSDYDWVSSHPNVSVSNTGAITLQDNPGGKV

IP31758 SGFNINSGFPTTGFKNTHFQLSPHGITGANSDYGWVSSHPNVSVSNTGAITLQDNPGGKV

*********************************.**************************

PB1 TITATWKHDSSKVFTYDFTLNYWVGLYSSTNLSWAQANASCINAGMRLPTNSEVSAGQDV

IP32953 TITATWKHDSSKVFTYDFTLNYWVGLYSSTNLSWAQANASCINAGMRLPTNSEVSAGQDV

YPIII TITATWKHDSSKVFTYDFTLNYWVGLYSSTNLSWAQANASCINAGMRLPTNSEVSAGQDV

IP31758 TITATWKHDSSKVFTYDFTLNYWVGLYSSTNLSWAQANASCINAGMRLPTNSEVSAGQDV

************************************************************

PB1 RGVGSLFGEWGNLNAYPSFPTAQIIWTSVDTNDFHIDTGLTHSASNVTLAYMCIK

IP32953 RGVGSLFGEWGNLNAYPSFPTAQIIWTSVDTNDFHIDTGLTHSASNVTLAYMCIK

YPIII RGVGSLFGEWGNLNAYPSFPTAQIIWTSVDTNDFHIDTGLTHSASNVTLAYMCIK

IP31758 RGVGSLFGEWGNLNAYPSFPTAQIIWTSVDTNDFHIDTGLTHSASNVTLAYMCIK

*******************************************************
