# Supplementary material for: Identification and characterisation of a novel adhesin Ifp in Yersinia pseudotuberculosis
Source: BMC Microbiol. 2011 Apr 28;11:85. doi: 10.1186/1471-2180-11-85 (PMC3102037; doi:10.1186/1471-2180-11-85)
Supplement: Additional file 2 — Growth curves from the temporal expression of Ifp and invasin assay. Within the Anthos Lucy1 combined photometer and luminometer, OD readings at 600 nm were taken at 30 minute intervals and used to construct these growth curves. Cultures were incubated at (A) 24°C (B) 28°C and (C) 37°C. [file 1471-2180-11-85-S2.PPT]

## Slide 1
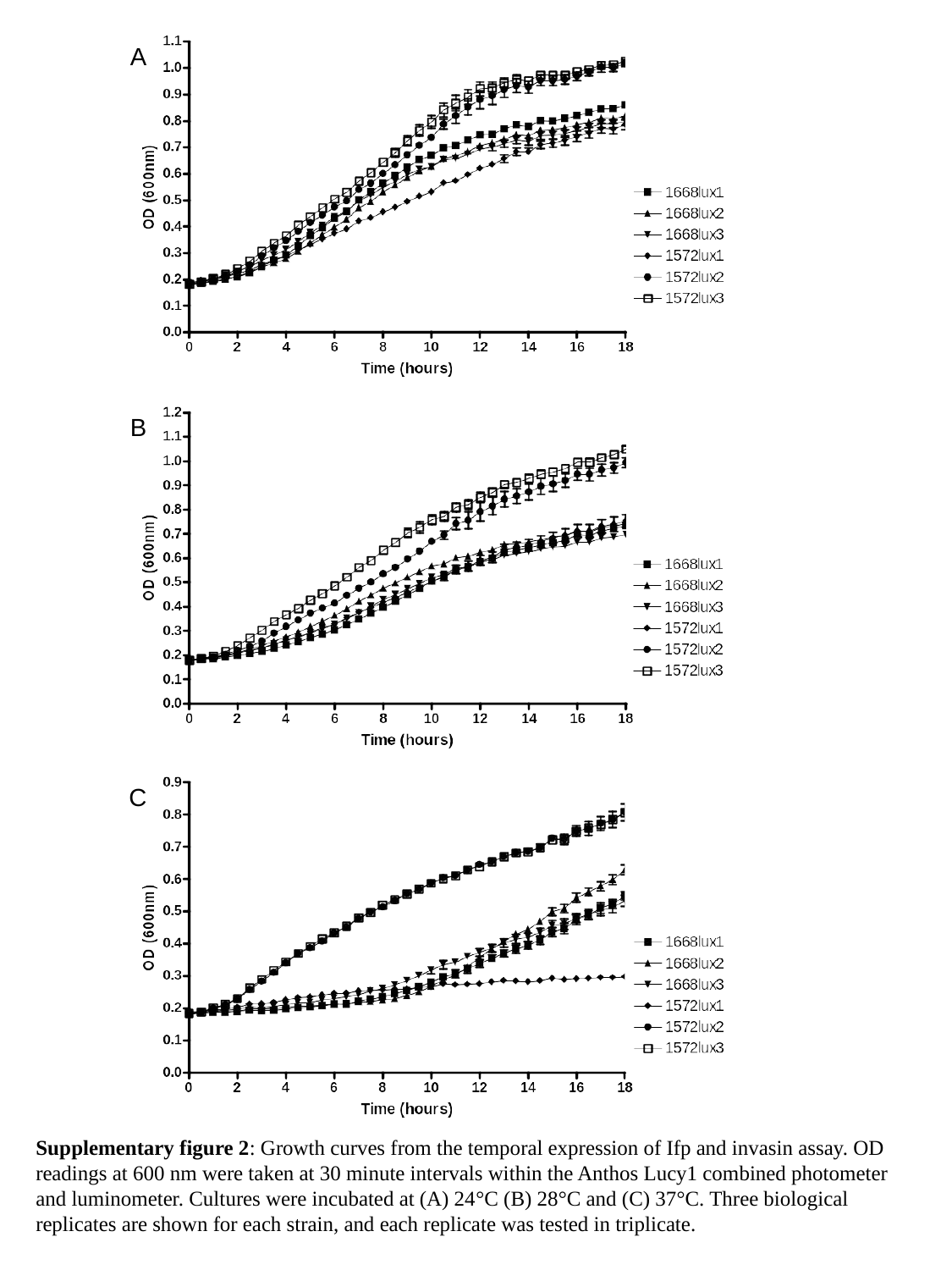

A
B
C
Supplementary figure 2: Growth curves from the temporal expression of Ifp and invasin assay. OD readings at 600 nm were taken at 30 minute intervals within the Anthos Lucy1 combined photometer and luminometer. Cultures were incubated at (A) 24°C (B) 28°C and (C) 37°C. Three biological replicates are shown for each strain, and each replicate was tested in triplicate.
